# Correspondence on “Venous thromboembolism prophylaxis regimen for patients undergoing deep inferior epigastric perforator flap breast reconstruction”

**DOI:** 10.1016/j.jpra.2026.04.004

**Published:** 2026-04-28

**Authors:** Preeti Lamba

**Affiliations:** Dr. D. Y. Patil Vidyapeeth (Deemed to be University), Pimpri, Pune, Maharashtra, India

*Dear Sir,*

I read with great interest the article by Kreul et al.^1^ titled “Venous thromboembolism prophylaxis regimen for patients undergoing deep inferior epigastric perforator flap breast reconstruction” published in JPRAS Open (2026;49:334–341). The authors have undertaken an important and commendable effort to evaluate a standardized, multimodal venous thromboembolism (VTE) prophylaxis regimen in patients undergoing DIEP flap breast reconstruction, a population recognized to be at increased thromboembolic risk due to prolonged operative duration, malignancy-associated hypercoagulability, and postoperative immobility. The study contributes meaningfully to existing literature by demonstrating a remarkably low VTE incidence following implementation of a comprehensive protocol incorporating preoperative, intraoperative and postoperative measures. Furthermore, the adoption of a uniform prophylaxis regimen irrespective of individual risk stratification highlights a pragmatic approach to perioperative care. These findings are in alignment with contemporary literature emphasizing the effectiveness of extended thromboprophylaxis strategies in reducing postoperative VTE events.^2^

Nonetheless, there are methodological considerations that deserve further discussion. One notable concern is the absence of a comparative analysis utilizing validated risk stratification tools such as the Caprini risk assessment model. While the authors suggest that a standardized approach may obviate the need for such models, omission of comparative evaluation restricts our understanding of the independent predictive value of uniform prophylaxis when adjusted for patient-specific risk factors. As demonstrated in recent studies, Caprini-based stratification continues to play a significant role in identifying high-risk patients undergoing DIEP flap reconstruction and guiding individualized thromboprophylaxis.^3^

Additionally, although the study reports a very low incidence of VTE, the occurrence of a delayed thrombotic event beyond the immediate postoperative period raises considerations regarding long-term surveillance and outcome attribution. Previous cohort studies have shown that a proportion of VTE events may occur after discharge, highlighting the importance of extended follow-up and risk-based prophylaxis duration in this patient population.^4^

In summary, the study by Kreul et al.^1^ provides valuable insights into standardized VTE prevention in patients undergoing DIEP flap breast reconstruction. Future research incorporating comparative study designs, multivariable risk stratification and extended follow-up may enhance generalizability and help guide individualized thromboprophylaxis strategies in reconstructive surgery.

## Funding

None.

## Ethical approval

None.

## Declaration of competing interest

None declared.
